# The lantibiotic nukacin ISK-1 exists in an equilibrium between active and inactive lipid-II binding states

**DOI:** 10.1038/s42003-018-0150-3

**Published:** 2018-09-25

**Authors:** Daisuke Fujinami, Abdullah-Al -Mahin, Khaled M. Elsayed, Mohammad R. Islam, Jun-ichi Nagao, Urmi Roy, Sabrina Momin, Takeshi Zendo, Daisuke Kohda, Kenji Sonomoto

**Affiliations:** 1 Division of Structural Biology, Medical Institute of Bioregulation, Kyushu University, Fukuoka, 812-8582 Japan; 2 Department of Bioscience and Biotechnology, Faculty of Agriculture, Graduate School, Laboratory of Microbial Technology, Kyushu University, Fukuoka, 812-8581 Japan; 3 Department of Functional Bioscience, Section of Infection Biology, Fukuoka Dental College, Fukuoka, 814-0175 Japan; 4 Present Address: Microbiology and Industrial Irradiation Division, Institute of Food and Radiation Biology, Atomic Energy Research Establishment, Ganakbari, Savar 1207, Dhaka, 1349 Bangladesh; 5 Present Address: Department of Microbiology, Faculty of Pharmacy, Misr International University, Cairo, 19648 Egypt; 6 Present Address: Department of Biochemistry and Molecular Biology, University of Dhaka, Dhaka, 1000 Bangladesh

## Abstract

The lantibiotic nukacin ISK-1 exerts antimicrobial activity through binding to lipid II. Here, we perform NMR analyses of the structure of nukacin ISK-1 and the interaction with lipid II. Unexpectedly, nukacin ISK-1 exists in two structural states in aqueous solution, with an interconversion rate on a time scale of seconds. The two structures differ in the relative orientations of the two lanthionine rings, ring A and ring C. Chemical shift perturbation induced by the titration of lipid II reveals that only one state was capable of binding to lipid II. On the molecular surface of the active state, a multiple hydrogen-bonding site formed by amino acid residues in the ring A region is adjacent to a hydrophobic surface formed by residues in the ring C region, and we propose that these sites interact with the pyrophosphate moiety and the isoprene chain of the lipid II molecule, respectively.

## Abstract (web summary)

Fujinami et al. show that an antimicrobial peptide Nukacin ISK-1 exists in an equilibrium between two states, only one of which can bind to the ISK-1’s target lipid-II, an important bacterial cell wall precursor. This study provides unexpected insights into the action modes of antibiotics.

## Introduction

**Fig. 1:**
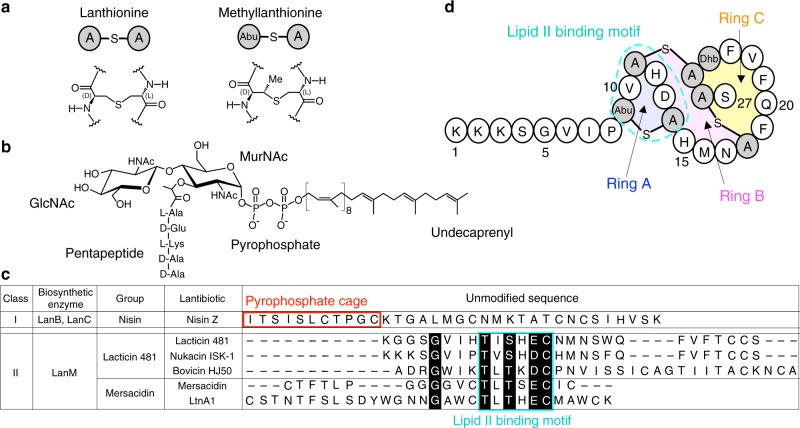
Primary structure of nukacin ISK-1 and lipid II. **a** Chemical structure of lanthionine and methyllanthione. **b** Chemical structure of lipid II. GlcNAc, N-acetylglucosamine; MurNAc, N-acetylmuramic acid. **c** Sequence alignment of the unmodified sequences of the class I and II lantibiotics. Highly conserved residues are highlighted in black. The lipid II pyrophosphate binding cage of the class I lantibiotics are enclosed by red lines. The lipid II binding motifs of the class II lantibiotics are enclosed by cyan lines. **d** Primary structure of nukacin ISK-1. Special regions are shaded and labeled: Ring A region (Abu9-Ala14), Ring B region (His15-Ala18), and Ring C region (Phe19-Ala26). Unusual amino acid residues are shown by shaded circles: Abu represents α-amino butyric acid; Dhb, dehydrobutyrine; Abu-S-A, methyllanthionine, and A-S-A, lanthionine, where “-S-“ denotes a monosulfide linkage

Owing to the emergence of antibiotic-resistant bacteria, such as vancomycin-resistant enterococci (VRE) and methicillin-resistant staphylococci (MRSA) in recent years, there is an imperative need for the development of novel antimicrobial agents^1^. Antimicrobial peptides possessing lanthionine or methyllanthionine residues are referred to as lantibiotics (Fig. 1a). The diversified and sophisticated modes of actions of lantibiotics have currently attracted significant research interests for finding plausible alternative antibiotics, in expectation of their future potential. For example, some lantibiotics have been used worldwide as a preservative in food industry^2^.

Lantibiotics are divided into four classes: I, II, III, and IV, based on their biosynthetic enzymes^3^. The biosynthesis of lantibiotics in two major classes, classes I and II, has been well characterized. Post-translational modifications of the class I lantibiotics are performed by two enzymes, LanB and LanC, which catalyze the dehydration of serine and threonine residues and the subsequent cyclization with a cysteine residue, respectively. In the class II lantibiotics, the same reactions are catalyzed by a single enzyme, LanM. The resultant monosulfide bridge structures, derived from a serine and cysteine pair and a threonine and cysteine pair, are referred to as lanthionine and methyllanthionine, respectively (Fig. 1a).

The representative of class I lantibiotics is nisin Z, which belongs to nisin group and consists of 34 amino acid residues. Nisin Z binds to lipid II, an essential intermediate in cell wall biosynthesis (Fig. 1b), to form pores in the cell membrane^4,5^. The solution structure of nisin Z in a complex with a water-soluble analog of lipid II revealed a binding site for a lipid II molecule, termed the ‘pyrophosphate cage’, locating close to the N-terminus^6^. Class II lantibiotics are more diverse than class I lantibiotics in terms of their amino acid sequences, and consist of two major groups and several minor groups^7^. The two major groups are referred to as lacticin 481 and mersacidin, of which members share a conserved lipid II binding motif^8^ (Fig. 1c).

The structures of lantibiotics in the mersacidin group were determined by X-ray crystallography and NMR^9–12^. For example, a structural model of the complex with a water soluble analog of lipid II was constructed for LtnA1, a component of the two-peptide lantibiotic lacticin 3147^12^. The model suggested that LtnA1 binds to the pyrophosphate moiety of lipid II via hydrogen bonds between the backbone amide groups in the lipid II binding motif. In contrast, the structures of lantibiotics in the lacticin 481 group are not well characterized. The NMR structural analyses of HJ50 and lacticin 481 were reported, but their structural convergences were not satisfactory, probably due to their dynamical properties^13,14^.

Nukacin ISK-1, which also belongs to the lacticin 481 group, is a 27-residue lantibiotics, containing two lanthionines, one methyllanthionine, and one dehydrobutyrine (Fig. 1d). *Staphylococcus warneri* ISK-1 isolated from an aged bed of fermented rice bran produces Nukacin ISK-1^15^. We previously performed the binding study with isothermal titration calorimetry to show the importance of the binding motif in ring A (Abu9-Ala14) for lipid II binding and inhibition of the cell wall biosynthesis^8^. We also reported that the N-terminal segment (Lys1-Pro8) was essential for their antimicrobial activity^16^. To understand the molecular basis of the mode of action, we determine the solution structure of nukacin ISK-1. Unexpectedly, two states exist with a slow interconversion rate, on a time scale of seconds. The conformations of the two states differ in the relative orientations of the two lanthionine rings. NMR titration experiments show that only one of the two states is capable of binding to lipid II. We also model the nukacin ISK-1 - lipid II complex with the aid of the chemical shift perturbation data. The model suggests that amino acid residues in and adjacent to ring A and the residues in ring C are involved in lipid II binding via intermolecular hydrogen bonding and hydrophobic interactions, respectively.

## Results

### Dynamic equilibrium between two states

**Fig. 2:**
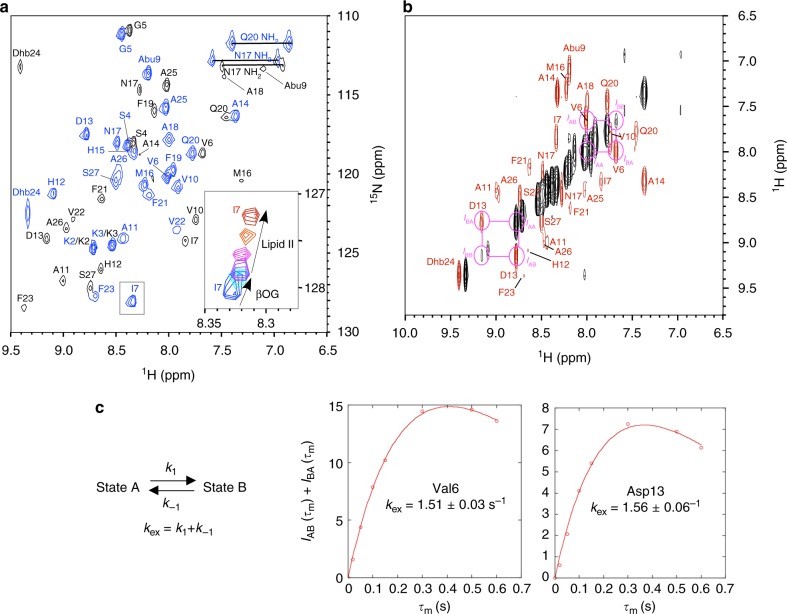
Intercoversion of two states of nukacin ISK-1. **a** ^1^H–^15^N HSQC spectrum of ^15^N, ^13^C-labeled nukacin ISK-1 in aqueous solution at 313 K. The ^1^H–^15^N cross peaks belonging to state A and state B are colored blue and black, respectively. The inset shows the movement of the cross peak of Ile7 of state A during the β-octylglucoside (βOG) titration from zero equivalent (blue) through 1 eq. (cyan) to 2 eq. of βOG (purple) to nukacin ISK-1, and during the successive lipid II titration from 0.25 eq. (magenta) through 0.5 eq. (orange) to 1.75 eq. (red) of lipid II to nukacin ISK-1. **b** Two-dimensional projection of 3D ^15^N-edited NOESY-HSQC spectrum of nukacin ISK-1. The diagonal peaks and EXSY cross peaks are colored black and red, respectively. The mixing time was 600 msec. The EXSY peaks used for the exchange rate analyses are enclosed by magenta circles. **c**. The mixing time (*τ*_m_) dependence of the sum of the EXSY cross peak intensities, *I*_AB_(*τ*_m_) + *I*_BA_(*τ*_m_), of Val6 and Asp13. The solid curves represent the nonlinear least-square fits to the expression *I*_AB_(*τ*_m_) + *I*_BA_(*τ*_m_) = (*I*_AA_(0) *p*_B_ + *I*_BB_(0)*p*_A_)[1-exp(-*k*_ex_*τ*_m_)]exp(-*R*_1_*τ*_m_), where the *p*_A_ and *p*_B_ are the relative populations for state A and state B, *k*_ex_ is the sum of the forward, *k*_1_, and reverse, *k*_-1_, kinetic rate constants for the interconversion between the two states, and *R*_1_ is the longitudinal relaxation rate of the amide protons

The ^1^H-^15^N HSQC spectrum of nukacin ISK-1 was recorded at 313 K in 10 mM sodium phosphate buffer, pH 6.0 (Fig. 2a). Unexpectedly, the HSQC spectrum revealed that the number of cross peaks was double the number of residues. Sequence-specific assignment of the ^1^H, ^13^C, and ^15^N resonances was performed using a standard suite of 3D triple resonance spectra of ^15^N, ^13^C-labeled nukacin ISK-1. Subsequently, exchanging cross-peaks were observed between the duplicated pairs of all of the residues, except for the N-terminal three residues, in the ^1^H–^1^H NOESY spectrum (Fig. 2b), indicating the existence of two states in the slow exchange regime on the chemical shift time scale. We refer to them as state A and state B (Fig. 2a). The peak intensity in the HSQC spectrum indicated that states A and B exist at a molar ratio of approximately 4:1 at 313 K in 10 mM sodium phosphate buffer, pH 6.0 (Supplementary Fig. 1).

We estimated the rate of the interconversion by the magnetization exchange spectroscopy (EXSY) experiment, of which pulse sequence was identical to 3D ^15^N-edited NOESY-HSQC. This method allows the determination of the exchange rate, *k*_ex_, in the time range of milliseconds to seconds. The EXSY cross peak intensities of well-resolved diagonal and cross peaks of Val6 and Asp13 were used to simulate the mixing-time dependency of the peak intensities (Fig. 2b). The non-linear fitting yielded the *k*_ex_ value of approximately 1.5 s^−1^ for the two residues (Fig. 2c and Table 1).

**Table 1:** Chemical exchange parameters of nukacin ISK-1^a^

Residue	*p*_A_	*p*_B_	*R*_1_ (s^−1^)	*k*_ex_ (s^−1^)
Val6	0.859	0.141	1.97 ± 0.04	1.51 ± 0.03
Asp13	0.883	0.117	2.28 ± 0.02	1.56 ± 0.06

^a^ From the EXSY experiment measured at 313 K, pH 6.0

### Two-step titration experiments

**Fig. 3:**
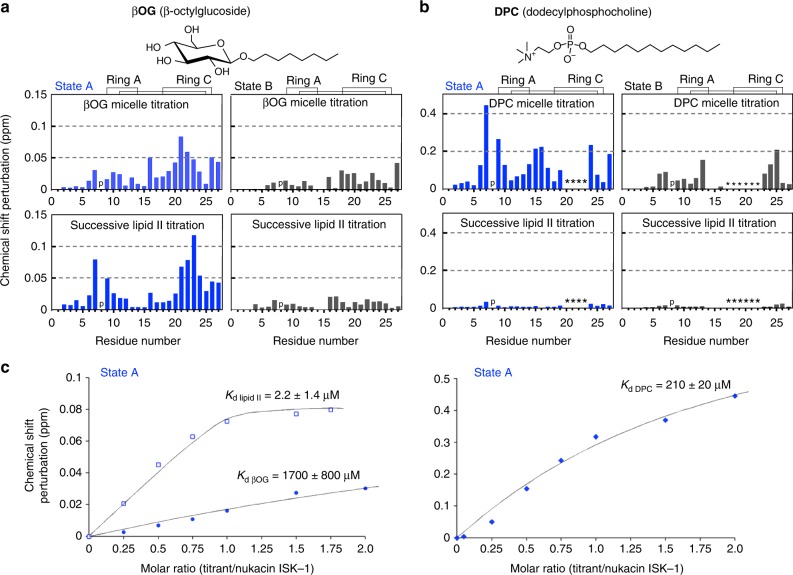
Chemical shift perturbations of the ^1^H–^15^N cross peaks of nukacin ISK-1 in two-step titrations. **a** Titration by the addition of β-octylglucoside (βOG) and the subsequent addition of lipid II. The chemical shift change of each backbone amide cross peak was calculated according to the equation, [(Δδ^1^H)^2^ + (Δδ^15^N/7)^2^]^1/2^ at saturating concentrations of the titrants. The titration reached saturation at a 1:2 molar ratio of nukacin ISK-1 to βOG micelle (assuming the aggregation number of 84), and at a 1:1.75 molar ratio of nukacin ISK-1 to lipid II monomer. **b** Titration by the addition of dodecylphosphocholine (DPC) and the subsequent addition of lipid II. The titration reached saturation at a 1:2 molar ratio of nukacin ISK-1 to DPC micelle (assuming the aggregation number of 52.5), and at a 1:3 molar ration of nukacin ISK-1 to lipid II monomer. The letter ‘p’ indicates the position of Pro8 in the nukacin ISK-1 sequence. The asterisks indicate the residues with cross peaks that became unobservable. **c** Titration curves of Ile7 in state A for the estimation of the dissociation constant of the state A nukacin ISK-1 for βOG micelle, lipid II, and DPC micelle. Best fit curves and the *K*_d_ values were calculated with the program xcrvfit

To investigate the binding of nukacin ISK-1 to lipid II, we performed two-step NMR titration experiments. In the first titration step, aliquots of a β-octylglucoside (βOG) solution were added to the nukacin ISK-1 solution in a stepwise manner. In the second titration step, the mixture of nukacin ISK-1 and βOG was transferred to a tube containing an aliquot of vacuum-dried lipid II. This process was repeated six times to gradually increase the concentration of lipid II. During the two-step titration (the inset of Fig. 2a), the cross peaks moved smoothly, and the resonance assignments of nukacin ISK-1 were easily transferred from the free state. The addition of βOG resulted in large chemical shift perturbations (CSPs) for several residues in the ring C region in state A (Fig. 3b). The subsequent lipid II addition resulted in large CSPs, not only in the residues in the ring C region but also in the residues in the ring A region and its N-terminally adjacent residue Ile7 in state A (Fig. 1b). These results suggested that hydrophobic residues in the ring C region were involved in the binding to both the hydrocarbon chain of βOG and the isoprene chain of lipid II, and the ring A region was involved in the specific interactions with lipid II. In contrast, the CSPs in state B were smaller than those in state A upon the titration of lipid II (Fig. 3a), suggesting that state B was incapable of binding to lipid II in the presence of βOG.

In the separate titration experiment, dodecyl phosphocholine (DPC) was used instead of βOG. The addition of DPC to nukacin ISK-1 solution resulted in the large CSPs in ring C at the initial step of titration (Supplementary Fig. 2), and disappearance of many cross peaks at the saturating concentration of DPC (Fig. 3b), supporting the idea that ring C is responsible for the hydrophobic interactions in the two states. We also observed large CSPs in the ring A region during the titration with DPC in state A. We inferred that the ring A region interacted with the phosphate group of DPC. The subsequent addition of lipid II in the presence of DPC did not induce further CSPs in the two states, indicating that nukacin ISK-1 embedded in DPC micelle could not bind to lipid II. DPC binding was reported for other pyrophosphate-targeting lantibiotics, such as mersacidin^11^ and nisin Z^17^.

Using the CSP titration curves of the ^1^H–^15^N cross peak of Ile7 (Fig. 3c), we estimated the binding affinity of the state A nukacin ISK-1 for βOG micelle, lipid II monomer, and DPC micelle. The binding for lipid II monomer was hundred-fold stronger than that for DPC micelle and thousand-fold stronger than that for βOG micelle.

### LiaRS reporter assay

**Fig. 4:**
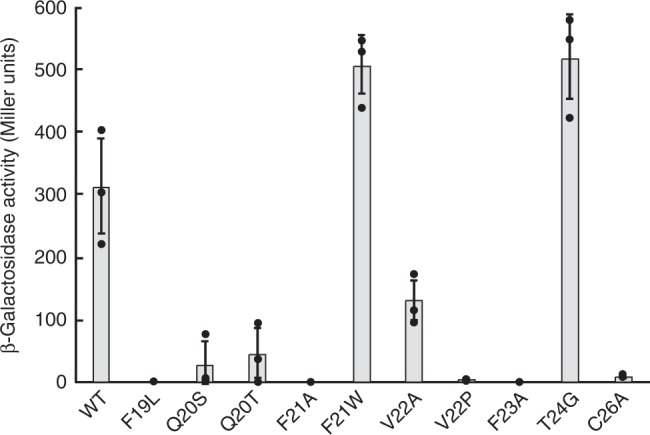
LiaRS (lipid II cycle interfering antibiotic response regulator and sensor) reporter assay of nukacin ISK-1 and its mutants. The β-galactosidase activity is correlated with the binding affinity of nukacin ISK-1 to lipid II. The averages of three independent experiments are shown. The error bars indicate the standard deviation (SD)

In order to evaluate the lipid II binding activity in vivo, we performed a LiaRS (lipid II cycle interfering antibiotic response regulator and sensor) reporter assay^18^. The upregulation of *lac*Z expression of β-galactosidase is monitored to measure the activation of the LiaRS two component system, induced by the binding of nukacin ISK-1 to lipid II. The LiaRS reporter assay indicated that the bulky hydrophobic residues, Phe19, Phe21, Val22, and Phe23, in ring C were indispensable for lipid II binding (Fig. 4). In addition, the side-chain carboxamide group of the polar Gln20 was involved in lipid II binding, because the replacement of Gln20 with Ser or Thr, which are also polar and uncharged amino acids, reduced the lipid II binding. The involvement of ring C in lipid II binding was further confirmed by the disruption of ring C, caused by the replacement of Cys26 with Ala. In accordance with the reduction of the LiaRS induction activity, these variants also showed reductions in their antimicrobial activities (Supplementary Table 1).

### Determination of solution structures

The interconversion between the two states made the NOE (nuclear Overhauser effect)-based structure determination complicated, due to the competition with the magnetization transfer through the chemical exchange mechanism. To overcome this difficulty, the equilibrium of the two states was shifted to one of the two states. Based on the ^1^H–^15^N HSQC peak intensities, we found that state A was dominant at a high temperature (326 K), whereas state B was dominant at a low temperature (283 K) (Supplementary Fig. 3). Thus, we performed two NOESY measurements to collect the state-specific NOE data. In the ^1^H–^1^H NOESY spectrum recorded at 283 K, the number of NOEs was sufficient to calculate the structure of state B, but in that measured at 326 K, the number of NOEs was insufficient for the meaningful calculation of the structure of state A. We found that the pH change from 6.0 to 3.5 and the addition of a sub-stoichiometric amount of dodecylphosphocholine (DPC, 0.05 micelle/nukacin ISK-1) were effective to increase the number of NOEs at 326 K. Upon addition of DPC, the chemical shifts were little affected, suggesting no significant conformational changes of nukacin ISK-1 in the two states.

**Fig. 5:**
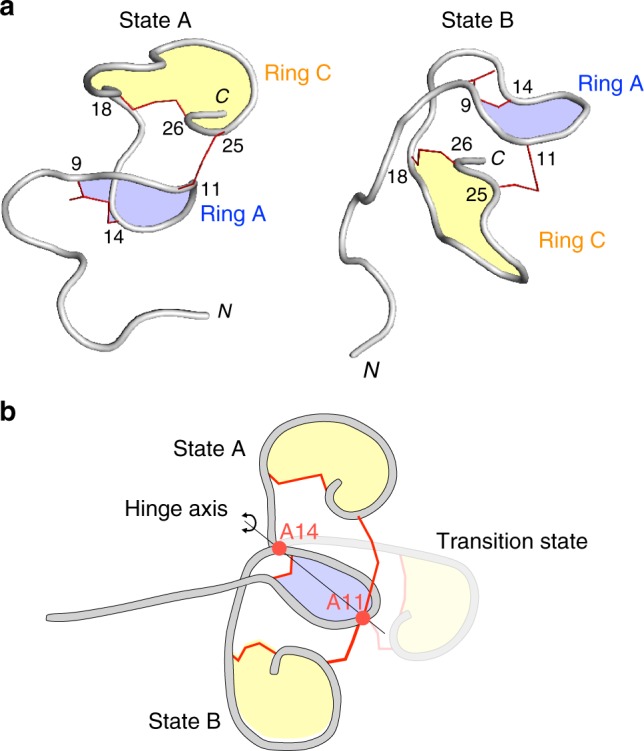
Cartoon representations of nukacin ISK-1 in state A and state B. **a** Ring A and ring C are colored blue and yellow, respectively. The monosulfide linkages of the two lanthionines and the one methyllanthionine are represented by thin red lines. **b** Proposed swing motion of ring C. The two residues, Ala11 and Ala14, act as a hinge

The structures of the two states were calculated using the program CYANA 2.1^19^ (Table 2 and Supplementary Fig. 4). The two structures differed in the orientation of ring C relative to ring A (Fig. 5a). Ring C is located above ring A in state A, whereas ring C is under ring A in state B. The comparison of the two structures suggested the swing motion of ring C, and the Ala11 and Ala14 act as a hinge during the swing motion (Fig. 5b). This drastic conformational transition explains the slow time scale interconversion between the two states in solution. Such slow interconversion processes between two states have been observed in protein mechanics, such as domain movement, topological conversion of secondary structures, and *trans-cis* peptide bond isomerization^20^. The *trans-cis* isomerization of the Ile7-Pro8 peptide bond could be the origin of the slow conversion. We measured the 1D ^1^H spectrum of a nukacin ISK-1 mutant, P8A, and found that the duplication of the amide proton peaks remained (Supplementary Fig. 5). Thus, the molecular basis of the two states of nukacin ISK-1 is not attributed to the *trans-cis* isomerization of a peptide bond.

**Table 2:** Statistics for the best 20 CYANA structures

Parameter (unit)	State A	State B
No. of structures		
Calculated	100	100
Selected	20	20
Target function (Å^2^)	0.98 ± 0.11	0.57 ± 0.12
No. of distance constraints		
Total NOE	130	152
Intra-residue NOE	40	47
Sequential NOE(|*i*-*j*| = 1)	60	45
Non-sequential NOE(|*i*-*j*| > 1)	30	60
Monosulfide linkage	3	3
No. of dihedral angle constraints		
Backbone (φ)	8	7
Upper distance violations (Å)		
rms^a^	0.018 ± 0.003	0.027 ± 0.004
Max	0.22	0.31
Van der Waals violations (Å)		
rms^a^	3.2 ± 0.4	1.5 ± 0.3
Max	0.28	0.28
Dihedral violations (degree)		
rms^a^	0.86 ± 0.25	0.59 ± 0.24
Max	2.33	1.45
rmsd^b^ of atomic coordinates (residues 1–27) (Å)		
Backbone (N, Cα, C)	1.75 ± 0.41	1.59 ± 0.55
Heavy atoms	2.58 ± 0.33	2.43 ± 0.55

^a^Root-mean-squared

^b^Root-mean-squared displacement

The slow processes were observed in previous NMR analyses of other members of the lacticin 481 group. van den Hooven et al. mentioned that the line broadening of the NMR signals of lacticin 481 was interpreted as the outcome of the interconversion between two low-energy structures^14^. The poor convergence of the NMR structure calculation of bovicin HJ50 was attributable to multiple conformers of ring C^13^. We propose that the dynamic equilibrium between multiple states would be a characteristic property of lantibiotics belonging to the lacticin 481 group.

### Structural insight into the interaction between nukacin ISK-1 and lipid II

**Fig. 6:**
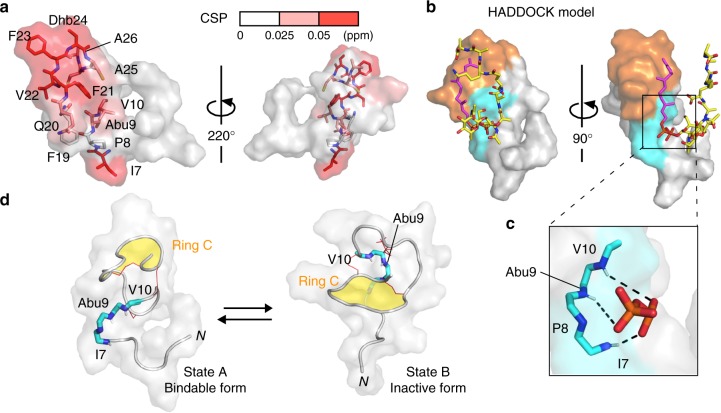
Proposed interaction between nukacin ISK-1 and lipid II. **a** Amino acid residues with substantial chemical shift perturbations upon binding to lipid II were mapped on the NMR-derived state-A structure. The residues were painted red (CSP > 0.050 ppm) and light red (CSP > 0.025 ppm). The side chains of the segment, Ile7-Val10, and those of the ring C residues are drawn in a stick representation. **b** Docking model of state-A nukacin ISK-1-lipid II complex generated by the HADDOCK calculation. The surface of the segment, Ile7-Val10, and the ring C residues are colored cyan and orange, respectively. The lipid II molecule is drawn in a stick representation with the isoprene chain in magenta, the pyrophosphate moiety in orange, and the MurNAc, GlcNAc, and peptapeptide in yellow. **c** Close-up view of the pyrophosphate-binding site in the HADDOCK model. The proposed intermolecular hydrogen bonds between the backbone amide groups of Ile7, Abu9, and Val10 and the pyrophosphate moiety of lipid II are indicated by black dashed lines. **d** Pyrophosphate-binding residues (cyan and blue sticks) in state A and in state B. In state B, the amide groups of Ile7, Abu9, and Val10 are oriented inward and masked by ring C (yellow), which prevents these amide groups from interacting with the pyrophosphate group of lipid II

We computationally docked a water-soluble lipid II analogue to the state-A structure based on the chemical shift perturbation (Fig. 3a), using the program HADDOCK2.2^21,22^. The large ^1^H–^15^N chemical shift perturbations (CSPs) were distributed on one side of the surface of the state A nukacin ISK-1 (Fig. 6a). The molecular surface formed by the hydrophobic side chains of ring C provides the hydrophobic surface for the interactions with the isoprene chain of lipid II (Fig. 6b, orange surface). Next to the hydrophobic surface, the backbone amide groups of Ile7, Abu9, and Val10 face outward, leading to intermolecular hydrogen bond formation with lipid II (Fig. 6c). This proposed interaction mode explains the inability of state B to bind to lipid II. In fact, in the state B structure, the amide groups of Ile7, Abu9, and Val10 are sequestered by ring C, thus inhibiting the interactions with the pyrophosphate moiety of lipid II (Fig. 6d).

To evaluate the proposed complex structure, we recorded ^1^H–^15^N heteronuclear NOE spectra of nukacin ISK-1 in the presence and absence of lipid II (Supplementary Fig. 6). The addition of lipid II increased the heteronuclear NOE values (i.e., the reduction of backbone flexibility) in the N-terminal region including Ile7, Abu9, and Val10, supporting the proposed interaction model. The proline mutants of Ile7 and Val10 remove the backbone amide hydrogen atoms. In our previous report, the replacement of Ile7 by Pro completely abolished the antimicrobial activity, whereas that of Val10 by Pro had no effects^23^. The latter result is unexplained at the moment. As for Abu9, the mutation destroys the monosulfide bridge and cannot be used for the verification.

The present data provide a model for the mode of action of nukacin ISK-1. Nukacin ISK-1 is first recruited to the membrane interface by binding to lipid II through the pyrophosphate motif in state A. Then, the ring C region of nukacin ISK-1 interacts with the isoprene chain of lipid II for deep insertion into the membrane. Eventually, the interactions of nukacin ISK-1 and lipid II leads to the inhibition of cell-wall biosynthesis^8^ and membrane destabilization^24^. We found that the inactive state B was a low temperature-induced state. State B may have a unique function, independent of the lipid II binding, such as a low-temperature sensor to recognize the environmental alternation and adapt to low-temperature environment^25^. The biological significance of state B of nukacin ISK-1 is an interesting issue to understand the mode and the control of the action of the antimicrobial activity, and to discover new functions of the lantibiotics belonging to the lacticin 481 group.

### Conclusion

We have reported the solution NMR study of nukacin ISK-1. NMR experiments revealed a dynamic equilibrium between two distinct states of nukacin ISK-1. The rate of interconversion between the two states was as slow as 1.5 s^−1^. The structures corresponding to the two states were determined at a high temperature condition (state A) and at a low temperature condition (state B). The titration experiments with the two-step addition of detergent micelle and lipid II revealed that only state A was able to bind to lipid II. The modeling study based on the chemical shift perturbations suggested two distinct lipid-II binding sites in the state A structure: one is the multiple hydrogen-bonding site formed by the backbone amide groups in and next to the ring A region for the pyrophosphate binding, and the other is a hydrophobic surface formed by hydrophobic residues in ring C for the isoprene chain binding. To our knowledge, our NMR structures are the first converged structures for the lacticin 481 group lantibiotics.

## Methods

### Preparation of nukacin ISK-1 and lipid II

Nukacin ISK-1 was purified according to the method reported by Aso et al.^26^. *Staphylococcous warneri* ISK-1 was grown overnight at 37 °C in Tryptic Soy Broth medium (Sigma-Aldrich, USA), supplemented with 0.6 % yeast extract (Nacalai Tesque, Japan). Nukacin ISK-1 in the culture supernatant was adsorbed to Amberlite XAD-16 resin (Sigma-Aldrich, USA), and washed out with 70 % isopropanol containing 0.1 % trifluoroacetic acid. Nukacin ISK-1 was purified by cation exchange chromatography and then reverse-phase HPLC. For stable isotope labeling of nukacin ISK-1, *S. warneri* ISK-1 was grown in 2 liters of ^15^N, ^13^C labeled CHL medium (Chlorella, Fukuoka, Japan). The labeled nukacin ISK-1 was purified in the same manner as the non-labeled nukacin ISK-1. The quantity of nukacin ISK-1 was determined with the BCA Protein Assay Kit-Reducing Agent Compatible (Thermo Scientific, USA). Lipid II was synthesized and purified as described in our previous report^8^. Purified lipid II was quantified by the amount of inorganic phosphate generated by perchloric acid treatment.

### NMR spectroscopy and resonance assignment

The 0.4-mL NMR sample for the resonance assignment and magnetization exchange spectroscopy contained 0.22 mM ^15^N, ^13^C-labeled nukacin ISK-1 and 1 μM sodium 2,2-dimethyl-2-silapentane-5-sulfonate (δ_H_ 0 ppm), dissolved in 10 mM sodium phosphate, pH 6.0, containing 10% ^2^H_2_O (v/v). NMR spectra were recorded on a Bruker Avance600 spectrometer equipped with a TXI cryoprobe, at 313 K. Sequence-specific backbone and side-chain assignments of the ^1^H, ^13^C, and ^15^N resonances were performed using ^1^H–^1^H TOCSY, ^1^H–^1^H NOESY, ^1^H-^13^C HSQC and a standard suite of 3D triple resonance NMR spectra, including HNHA, HNCACB, CBCA(CO)NH, HNCA, HN(CO)CA, HNCO, HN(CA)CO, HAHB(CO)NH, C(CO)NH, and H(CCO)NH^27^. The magnetization exchange spectroscopy was performed using a 3D ^15^N NOESY-HSQC pulse sequence with mixing times (*τ*_m_) of 20, 50, 100, 150, 300, 500, and 600 ms. For the structure calculation of state A, ^1^H-^1^H NOESY with a mixing time of 300 ms was measured at 328 K, using 13 mM non-labeled nukacin ISK-1 dissolved in 20 mM Gly-HCl, pH 3.5, and 34 mM (monomer concentration) DPC, containing 10% ^2^H_2_O (v/v). For the structure calculation of state B, ^1^H–^1^H NOESY (*τ*_m_ = 300 ms) was measured at 283 K, using 1.1 mM non-labeled nukacin ISK-1 dissolved in 20 mM sodium phosphate, pH 6.0, containing 10% ^2^H_2_O (v/v). The ^1^H–^15^N heteronuclear NOE spectra of nukacin ISK-1 were recorded in the absence and presence of lipid II at 313 K. Three-second weak presaturation was applied on- or off-resonance of the amide proton frequency^28^. NMR data were processed and displayed with the program nmrPipe/nmrDraw, version 3.0^29^.

### Interconversion rate determination

In a dynamic equilibrium between state A and state B, the intensities of the auto peaks, *I*_AA_(*τ*_m_) and *I*_BB_(*τ*_m_), and those of the cross peaks, *I*_BA_(*τ*_m_) and *I*_AB_(*τ*_m_), are governed by the equations:

$$I_{\mathrm{{AA}}}\left( {\tau _{\mathrm{m}}} \right) = I_{\mathrm{{AA}}}\left( 0 \right)\left[ {p_{\mathrm{A}} + p_{\mathrm{B}}\exp \left( { - k_{\mathrm{{ex}}}\tau _{\mathrm{m}}} \right)} \right]\exp \left( { - R_1\tau _{\mathrm{m}}} \right)$$

$$I_{\mathrm{{BB}}}\left( {\tau _{\mathrm{m}}} \right) = I_{\mathrm{{BB}}}\left( 0 \right)\left[ {p_{\mathrm{B}} + p_{\mathrm{A}}\exp \left( { - k_{\mathrm{{ex}}}\tau _{\mathrm{m}}} \right)} \right]\exp \left( { - R_1\tau _{\mathrm{m}}} \right)$$

$$I_{\mathrm{{BA}}}\left( {\tau _{\mathrm{m}}} \right) = I_{\mathrm{{AA}}}\left( 0 \right)p_{\mathrm{B}}\left[ {1 - {\mathrm{exp}}\left( { - k_{\mathrm{{ex}}}\tau _{\mathrm{m}}} \right)} \right]\exp \left( { - R_1\tau _{\mathrm{m}}} \right)$$

$$I_{\mathrm{{AB}}}\left( {\tau _{\mathrm{m}}} \right) = I_{\mathrm{{BB}}}\left( 0 \right)p_{\mathrm{A}}\left[ {1 - {\mathrm{exp}}\left( { - k_{\mathrm{{ex}}}\tau _{\mathrm{m}}} \right)} \right]\exp \left( { - R_1\tau _{\mathrm{m}}} \right)$$

where the *p*_A_ and *p*_B_ are relative populations for state A and state B, *k*_ex_ is the sum of the forward, *k*_1_, and reverse, *k*_−1_, kinetic rate constants for the interconversion between the two states, and *R*_1_ is the longitudinal relaxation rate of the amide protons^30^. The *p*_A_ and *p*_B_ were obtained from the peak intensities in the ^1^H–^15^N HSQC spectrum. The *R*_1_ values are assumed to equal for the two states. The total intensity, *I*_AB_(*τ*_m_) + *I*_BA_(*τ*_m_), as a function of mixing time, *τ*_m_, were fitted to the sum of the third and fourth equalities to obtain *R*_1_ and *k*_ex_. The non-linear fitting was performed using KaleidaGraph 4.0 (Synergy Software, USA).

### Two-step titration experiments

In the first titration step, aliquots of 1 M *n*-octyl-*d*_17_-β-D-glucopyranoside (βOG-*d*_17_, CDN Isotopes, Quebec, Canada) dissolved in 10 mM sodium phosphate, pH 6.0, were added to the nukacin ISK-1 solution (0.22 mM) in a stepwise manner at nukacin ISK-1:βOG-*d*_17_ micelle molar ratios of 1:0, 1:0.25, 1:0.5, 1:0.75, 1:1, 1:1.5, and 1:2. The concentration of the βOG-*d*_17_ micelle was calculated by assuming the aggregation number of 84. The chemical shift perturbation was almost saturated at the molar ratio of 1:2, in which the final βOG-*d*_17_ monomer concentration was 35.2 mM. In the second titration step, lipid II in chloroform/methanol/water was divided into separate tubes and dried to thin film in a centrifugal vacuum concentrator (SpeedVac, Thermo Scientific, USA). The mixture solution of nukacin ISK-1 and βOG-*d*_17_ micelle was put in one of the tubes and then rigorously sonicated for the complete dissolution of the lipid II film. This process was repeated six times to adjust the nukacin ISK-1: lipid II monomer molar ratios of 1:0, 1:0.25, 1:0.5, 1:0.75, 1:1, 1:1.5, and 1:1.75. The chemical shift perturbations were saturated when the molar ratio of nukacin ISK-1: lipid II reached 1:1.75. For the two-step titration with dodecyl phosphocholine (DPC) detergent, aliquots of 100 mM perdeuterated DPC-*d*_38_ (Cambridge Isotope Laboratories, USA) dissolved in 10 mM sodium phosphate, pH 6.0, were added to the nukacin ISK-1 solution (0.125 mM) in a stepwise manner at nukacin ISK- 1:DPC-*d*_38_ micelle molar ratios of 1:0, 1:0.25, 1:0.5, 1:0.75, 1:1, 1:1.5, and 1:2. The concentration of the DPC-*d*_38_ micelle was calculated by assuming the aggregation number of 52.5. The chemical shift perturbation was almost saturated at the molar ratio of 1:2, at which the final DPC monomer concentration was 18.6 mM. The cross peaks corresponding to the segment Gln20-Dhb24 in state A, and the segment Met16-Phe23 in state B, disappeared when the molar ratio of nukacin ISK-1: lipid II reached 1:0.5. The lipid II titration in the presence of DPC was performed in the same manner as the two-step titration experiment with βOG and lipid II. The titration curves were analyzed with the program xcrvfit, ver. 4.0.12 (http://www.bionmr.ualberta.ca/bds/software/xcrvfit/) to calculate the dissociation constants.

### Structure determination

The NMR structures of nukacin ISK-1 were calculated with CYANA 2.1^19^. The distance restraints were derived from 2D ^1^H-^1^H NOESY spectra, and the upper distance limits were uniformly set to 5.5 Å. For connecting monosulfide linkages, an upper-limit distance restraint (2.0 Å) was applied between the sulfur and C_β_ atoms of lanthionine and metyllanthionine. The ϕ angle restraints were set to −120° ± 40° and 120° ± 40° for L-amino and D-amino acid residues, respectively, when a large ^3^*J*_HNHA_ coupling constant (>8 Hz) was observed in 3D HNHA spectrum^31^. The configuration of the peptide bond between Ile7 and Pro8 was determined to be *trans* by the ^13^C_β_ and ^13^C_γ_ chemical shift values for the two states, and thus the peptide bond was fixed to *trans* in the CYANA calculations. The configuration of the C_α_–C_β_ double bond in Dhb24 was confirmed to be *cis* by a strong intraresidual NOE between the HN and H_γ_ resonances. The CYANA library files of non-standard amino acid residues (D-Ala, D-Abu, and Dhb) were converted from the mmCIF entries of the PDB Chemical Component Dictionary (DAL.cif, ABA.cif, and DBU.cif) by the program CYLIB^32^. The coordinates of atoms for state A and state B were deposited in the Protein Data Bank (PDB IDs 5Z5Q and 5Z5R), and the chemical shift tables were deposited in the BioMagResBank (IDs 36157 and 36158). The figure generation was performed with PyMOL, version 1.5 (Schrödinger).

### Model building of the nukacin ISK-1–lipid II complex

A water-soluble lipid II analogue was computationally docked to the state-A nukacin ISK-1 structure using the program HADDOCK2.2^21,22^. The atomic coordinates of the water-soluble lipid II analogue were obtained from PDB with entry 1WCO^6^. Ambiguous interaction restraints (AIRs) were generated based on the distribution of the chemical shift perturbation data. Residues 7, 9, 10, and 23 of nukacin ISK-1 were defined as active residues, and residues 6, 8, 21, and 22 were set passive. The pyrophosphate group and the isoprene chain of lipid II were defined active, and the rest was set passive. We added two intermolecular hydrogen bond restraints: one restraint is between O2 of the phosphate group on the sugar side in lipid II and HN of Ile7 in nukacin ISK-1. The other restraint is between O2 of the phosphate group on the isoprene side in lipid II and HN of Val10 in nukacin ISK-1. The N-terminal segment, Lys1-Val6, of nukacin ISK-1 and the isoprene chain of lipid II were treated as fully flexible regions in the refinement stage. The option for the removal of non-polar hydrogens was turned off. All other parameters were the default settings. The one with the lowest HADDOCK score was selected as the best HADDOCK model for further discussion.

### Determination of antimicrobial activity

The nukacin ISK-1 variants were generated by a saturation mutagenesis technique using NNK degenerate codons in our previously study^23^. The mutated nukacin ISK-1 peptides were purified from the culture supernatant of recombinant *Lactococcus lactis* NZ9000 strains, according to the procedure described by Aso et al.^26^. The antimicrobial activities of nukacin ISK-1 and its ring C variants were determined by the spot-on-lawn method^33^. Lactobacillus agar AOAC (BD, Sparks, MD) was overlaid on MRS agar medium (Oxoid, Basingstoke, UK) with the specific indicator strain (*Lactobacillus sakei* subsp. *sakei* JCM 1157 ^T^), and a series of two-fold dilutions of the peptides were spotted onto the surface of the medium. After an overnight incubation, the minimum inhibitory concentration (MIC) was determined as the lowest peptide concentration causing inhibition of visible bacterial growth.

### LiaRS reporter assay

The *liaI* promoter induction was performed using the microtiter plate bioassay described previously^34,35^. Briefly, an overnight culture of the *Bacillus subtilis* BFS 2470 strain was diluted in TY/0.3 M NaCl medium to an OD_600_ of 0.1 and incubated at 37 °C for 2.5 h. The culture was dispensed into a 96-well microtiter plate, and then nukacin ISK-1 or its ring C variants (1 μg/ml) were added. The microtiter plate was incubated at 37 °C for 1 h. After cooling, cells were harvested by centrifugation at 2800 *g* for 10 min and the supernatant was discarded. Cells were resuspended in 100 μl Z-buffer (6 mM Na_2_HPO_4_, 40 mM NaH_2_PO_4_, 10 mM KCl, 1 mM MgSO_4_, pH 7.0). Then, the optical density was determined at 600 nm using a plate reader. Cells were permeabilized using toluene. β-galactosidase reaction was initiated by the addition of 35 μl *o*-nitrophenyl-β-D-galactopyranoside (4 mg/ml in Z-buffer) at 28 °C and stopped by the addition of 80 μl of 1 M Na_2_CO_3._ Then, the absorbance at 420 and 550 nm were measured. The β-galactosidase activity was calculated in Miller units^36^.

## Electronic supplementary material

Supplementary Information

## Data Availability

The coordinates of atoms for nukacin ISK-1 in active and inactive state were deposited in the Protein Data Bank (PDB IDs 5Z5Q and 5Z5R), and the chemical shift tables were deposited in the BioMagResBank (IDs 36157 and 36158). All the other data generated or analyzed during this study are included in this published article and its Supplementary Information.
