# Supplementary material for: The lantibiotic nukacin ISK-1 exists in an equilibrium between active and inactive lipid-II binding states
Source: Commun Biol. 2018 Sep 25;1:150. doi: 10.1038/s42003-018-0150-3 (PMC6156582; doi:10.1038/s42003-018-0150-3)
Supplement: Supplementary file 1 — Supplementary Information [file 42003_2018_150_MOESM1_ESM.pdf]

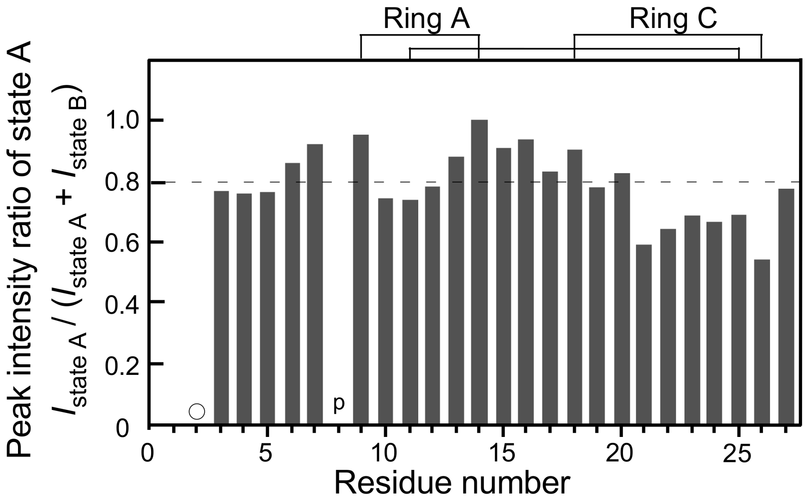

Supplementary Figure 1

The fraction of state A was estimated from the peak intensity ratio of state A, and plotted as a function of the nukacin ISK-1 residue number. The peak intensity was estimated from the peak height of the ^{1}H-^{15}N HSQC cross peaks at 313 K at pH 6.0. The open circle indicates that the peak intensity of Lys2 was not determined because of the overlap of the cross peaks of the two states. The letter ‘p’ in the panel indicates the position of Pro8 in the nukacin ISK-1 sequence.

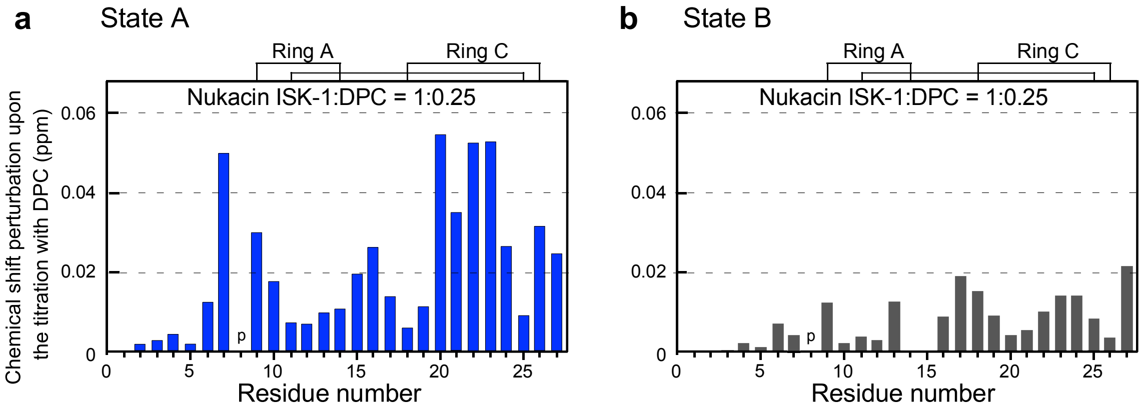

Supplementary Figure 2

Chemical shift perturbation of the ^{1}H-^{15}N cross peaks of nukacin ISK-1 at 1:0.25 molar ratio of nukacin ISK-1 to dodecylphosphocholine (DPC) micelle. Note that the perturbation at the saturated concentration of DPC micelle is shown in Figure 3*b*. The addition of DPC to nukacin ISK-1 produced significant chemical shift perturbations in the segment Ile7-Val10 and the ring C of state A, but not in those of state B. The letter ‘p’ in the panel indicates the position of Pro8 in the nukacin ISK-1 sequence.

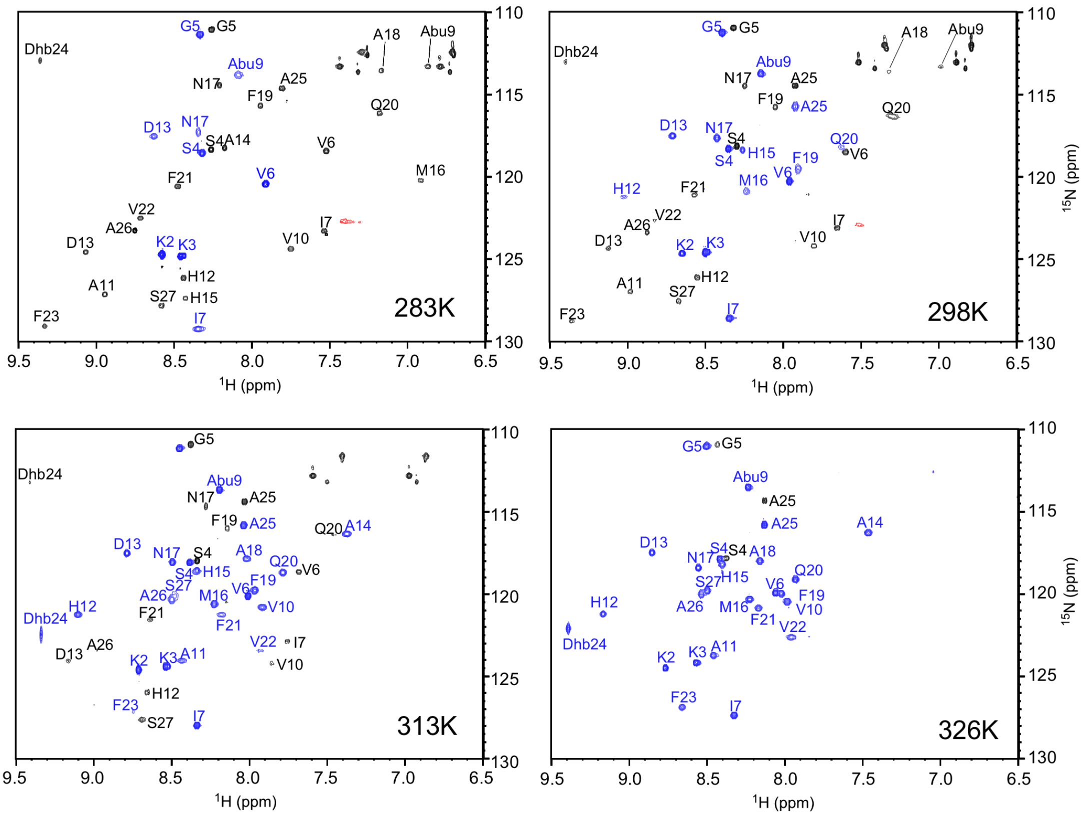

Supplementary Figure 3

Temperature effects on the equilibrium of the two states of nukacin ISK-1. The ^{1}H-^{15}N HSQC spectra of nukacin ISK-1 recorded at 283 K, 298 K, 313 K and 326 K are shown. The cross peaks in state A are colored blue, and those in state B are colored black. Red peaks (negative intensity due to aliasing along the ^{15}N axis) are derived from the protonated form of the ε-amino group of lysine residues.

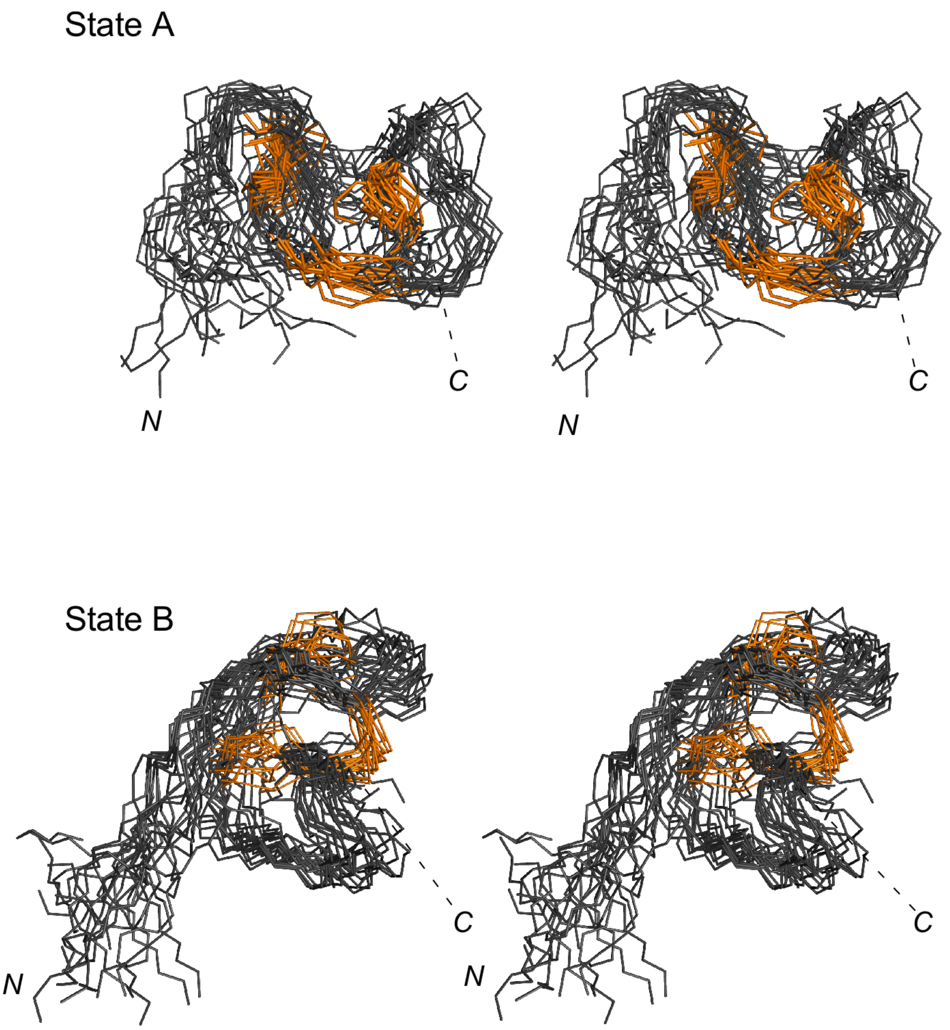

Supplementary Figure 4

Stereo views of the calculated structure ensembles of nukacin ISK-1 in state A and state B. The 20 selected structures were superimposed for each state. The monosulfide linkages of the two lanthionines and one methyllanthionine are depicted as brown sticks.

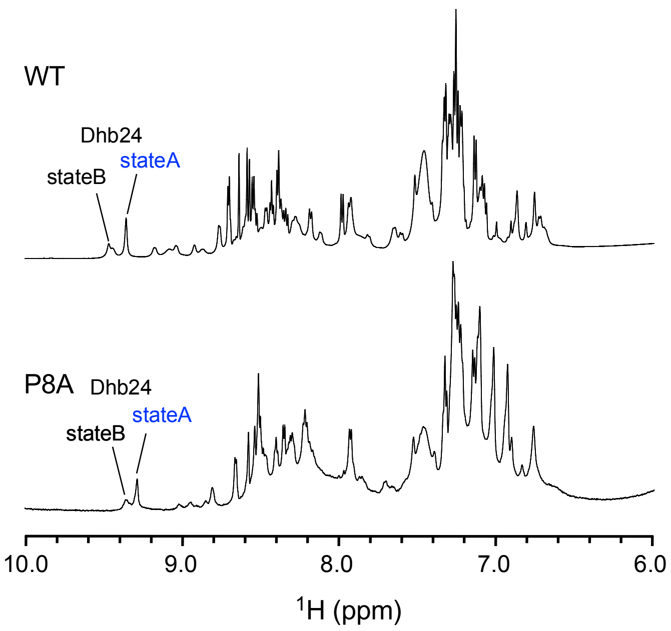

Supplementary Figure 5

One-dimensional ^{1}H spectra of WT and P8A mutant of nukacin ISK-1. The P8A spectrum contained two amide proton peaks of Dhb24, indicating the existence of two states, as is the case with the wild type of nukacin ISK-1.

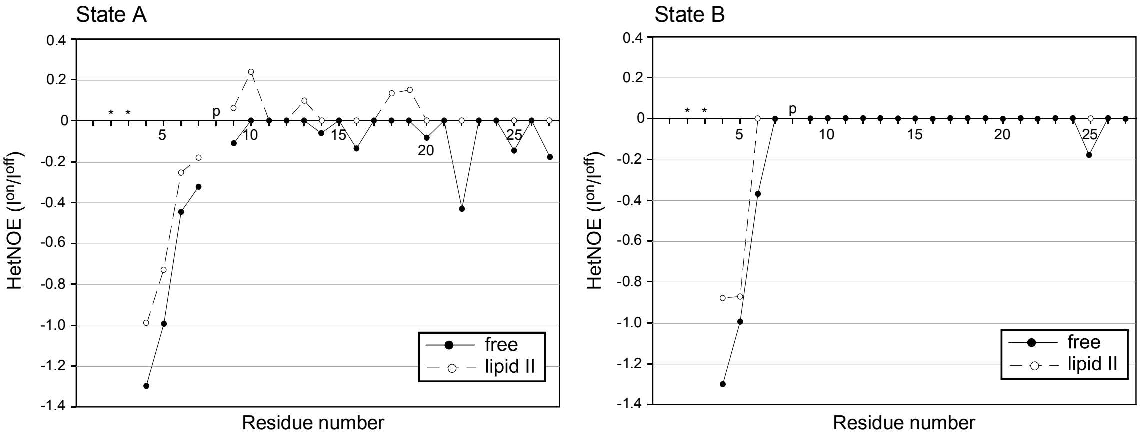

Supplementary Figure 6

The [1]H-^{15}N heteronuclear NOEs of nukacin ISK-1 in the presence (open circles) and the absence of lipid II (filled circles) at 313K. The asterisks indicate that the heteronuclear NOEs for Lys2 and Lys3 were not obtained due to peak overlaps of the two states. The letter ‘p’ in the panel indicates the position of Pro8 in the nukacin ISK-1 sequence.

Supplementary Table 1

Antibacterial Activity of Nukacin ISK-1 and the Ring C Mutants.

Nukacin ISK-1	Position	MIC (μg/ml)[a]
Wild type		3.12
F19L	Ring C mutant	25
Q20S	Ring C mutant	50
Q20T	Ring C mutant	50
F21A	Ring C mutant	>50
F21W	Ring C mutant	6.25
V22A	Ring C mutant	50
V22P	Ring C mutant	>50
F23A	Ring C mutant	>50
T24G	Dehydrated Thr	6.25
C26A	Ring C disrupter	>50

[a]Antibacterial activity against *Lactobacillus sakei* subsp. *sakei* JCM 1157^T was measured twice for each mutant. The minimum inhibitory concentration (MIC) was determined as the lowest peptide concentration causing inhibition of visible bacterial growth.
